# The Use of Platelet-Rich Fibrin in Combination With Biphasic Calcium Phosphate in the Treatment of Bone Defects: A Histologic and Histomorphometric Study^☆^

**DOI:** 10.1016/j.curtheres.2013.05.002

**Published:** 2013-12

**Authors:** Nilüfer Bölükbaşı, Sinem Yeniyol, Merva Soluk Tekkesin, Kemal Altunatmaz

**Affiliations:** 1 Department of Oral Implantology, Faculty of Dentistry, Istanbul University, Istanbul, Turkey; 2 Institute of Oncology, Department of Tumor Pathology, Istanbul University, Istanbul, Turkey; 3 Department of Surgery, Faculty of Veterinary Medicine, Istanbul University, Istanbul, Turkey

**Keywords:** biphasic calcium phosphate, bone augmentation, platelet-rich fibrin

## Abstract

**Background:**

Platelet-rich fibrin (PRF) is a leukocyte and platelet concentrate containing many growth factors. Its potential for hard tissue augmentation as a sole grafting material or in combination with other grafting materials has been investigated in many studies.

**Objective:**

The aim of this histologic study was to evaluate the efficacy of PRF mixed with biphasic calcium phosphate (BCP) on bone regeneration in surgically created bone defects.

**Methods:**

Defects 5 mm in diameter were created in both tibias of 6 sheep. The defects were left empty or grafted with BCP, PRF, or BCP+PRF. Animals were killed at 10, 20, and 40 days. The specimens underwent histologic and histomorphometric analysis.

**Results:**

None of the groups displayed any signs of necrosis. Inflammation was observed in all groups at 10 days; 2 specimens of PRF+BCP and all empty defects showed inflammatory cell infiltration at 20 days. During the 40-day evaluation period, the PRF+BCP group showed the highest ratios of new bone. The other 3 groups showed statistically similar results. In the BCP and PRF+BCP groups, the residual graft ratios were decreased at consecutive time intervals. The difference between the 2 groups was not statistically significant during follow-up.

**Conclusions:**

The current study revealed a histomorphometric increase in bone formation with the addition of PRF to BCP in surgically created defects in sheep tibia.

## Introduction

Platelet-rich fibrin (PRF) is an autologous fibrin matrix. It was described by Choukroun et al^1^ specifically for oral and maxillofacial surgery applications. PRF can be classified as a second-generation platelet concentrate because it contains leukocytes and does not require an anticoagulant.^2–4^ PRF use is a simple technique. Venous blood is collected in 10-mL dry tubes and centrifuged for 12 minutes at 2700 rpm (∼400*g*). After centrifugation, 3 layers are obtained: red blood cells at the bottom, acellular plasma at the top (platelet-poor plasma), and PRF between the 2 layers. It was shown that, after centrifugation, ∼97% of platelets and 50% of leukocytes of the original blood volume were concentrated in the PRF.^5^ Previous studies showed the slow release of growth factors such as transforming growth factor β1, platelet-derived growth factor β, and vascular endothelial growth factor, especially during the first 7 days.^6,7^. Zumstein et al^8^ also reported that this release continued with the decrease up to 28 days. PRF can be used as the sole biomaterial^9–21^ or combined with different bone substitutes.^11,22–27^ The use of PRF in conjunction with grafting materials would accelerate bone regeneration.

Biphasic calcium phosphate (BCP) is a commonly used synthetic bone substitute comprising less soluble hydroxyapatite (HA) and more soluble β-tricalcium phosphate (β-TCP). It is a biocompatible, osteoconductive, and cost-effective biomaterial. The main advantage of BCP is that its chemical composition is similar to that of apatite in biological bone.^28–30^ The hard particles of HA support the bulk of the graft material, and β-TCP increases the replacement of its degradation products with blood vessels and lamellar bone.^31^

Although there are several previous reports on the use of PRF in bone augmentation, none investigated the combination of PRF+BCP. The aim of this experimental study was to evaluate the inflammatory features and effectiveness of PRF+BCP combination to improve and accelerate bone regeneration in surgically created bone defects in sheep.

## Methods

### Animal model and surgical procedure

**Fig. 1:**
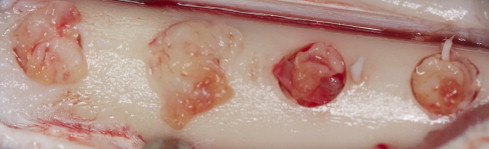
Clinical view of surgically created defects. The first 2 defects were grafted with biphasic calcium phosphate mixed with platelet-rich fibrin (PRF) at a 1:1 ratio and covered with PRF membrane. The other 2 defects were grafted with only PRF.

**Fig. 2:**
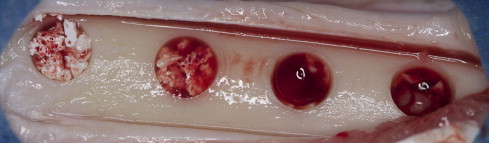
Clinical view of surgically created defects. The first 2 defects were grafted with biphasic calcium phosphate, and the other 2 defects were left empty.

The study was approved by the Animal Ethical Committee of Istanbul University (no. 2011-91). All procedures were conducted in accordance with the Istanbul University ethical guidelines for the treatment and welfare of experimental animals. The study was conducted at the Istanbul University, Faculty of Veterinary Medicine, Department of Surgery between January 2012 and February 2012. The study included 6 adult male sheep (age, 2–3 years; weight, 40–50 kg).The animals were fasted for 24 hours preoperatively. Ceftriaxone sodium 22 mg/kg (1 g) (Iesef, Ulugay, Turkey) was administered intramuscularly to reduce the risk of postoperative infection. Surgical procedures were performed under general anesthesia under sterile conditions. Xylazine⁎ (0.2–0.5 mg/kg IM) was given as premedication. Preanesthesia with 22 mg/kg IV ketamine hydrochloride† was administered. The animals were then intubated. General anesthesia was achieved using an intravenous injection of pentobarbital and maintained with isoflurane 1.5%–2%, administered through the endotracheal tube. Both tibias were shaved and cleansed with 10% povidone iodine and 70% alcohol swabs before manipulation. Skin and periosteal incisions were created separately, and the tibias were exposed. In each tibia, 4 monocortically defects 5 mm in width and depth were prepared with a trephine burr under saline solution irrigation at 1500 rpm. A distance of 5 mm was left between each defect. The first 2 defects on the right side of the tibia were grafted with BCP (HA-to-TCP ratio: 60:40) (4Bone, MIS, Tel-Aviv, Israel) mixed with PRF (1:1 ratio) and covered with PRF membrane (Figure 1). The other 2 defects in the right tibia were grafted with only PRF. The defects in the left tibia were left empty or grafted with only BCP (Figure 2). After obtaining adequate hemostasis, the periosteum was closed with 4-0 resorbable suture (Vicryl, Ethicon, Turkey), and the skin was closed with a skin stapler. The tibias were fastened down to avoid fracture. The animals were given ceftriaxone sodium 22 mg/kg twice daily (1 g, IM; Iesef) as an antibiotic for 5 days postoperatively. The animals were placed in separate cages in a standard environment to allow the animals to live and fed a standard diet. During the study period, the animals were examined for leg fractures, infection, and adverse reactions.

The animals were killed at 10, 20, and 40 days (2 animals at each time interval) using an overdose of sodium pentothal (30 mg/kg, IV, Abbott, Istanbul, Turkey). The tibias were carefully dissected free from soft tissues, and hard-tissue samples were transferred into 10% buffered-formalin solution.

### PRF preparation

**Fig. 3:**
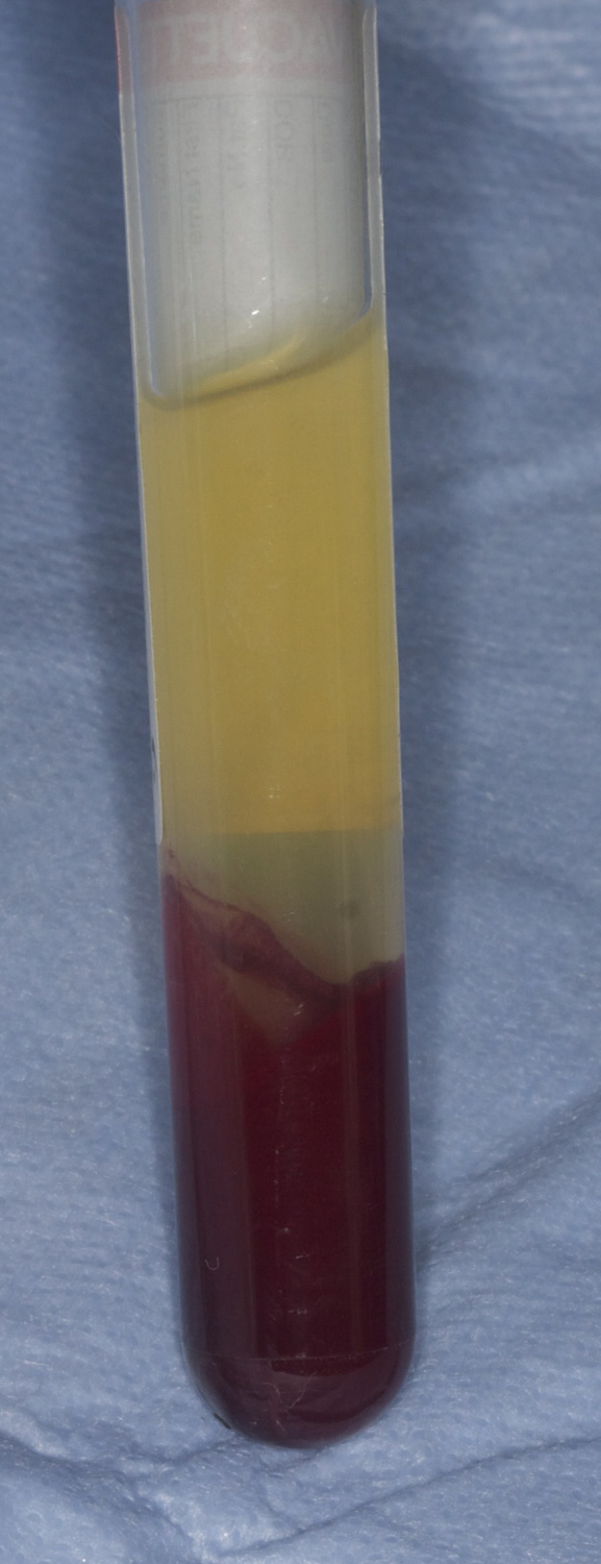
Three layers were obtained after centrifugation at 400*g* for 12 minutes. The fibrin clot (platelet-rich fibrin) was concentrated in the middle of the tube, between the red corpuscles at the bottom and a cellular plasma (platelet poor plasma) at the top.

**Fig. 4:**
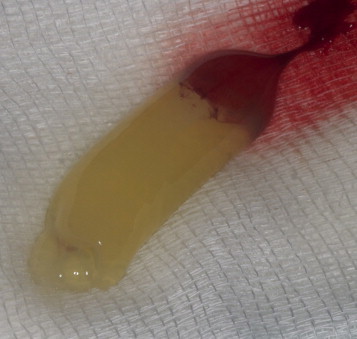
Fibrin clot (platelet-rich fibrin) clot separated from red corpuscles by tweezers.

**Fig. 5:**
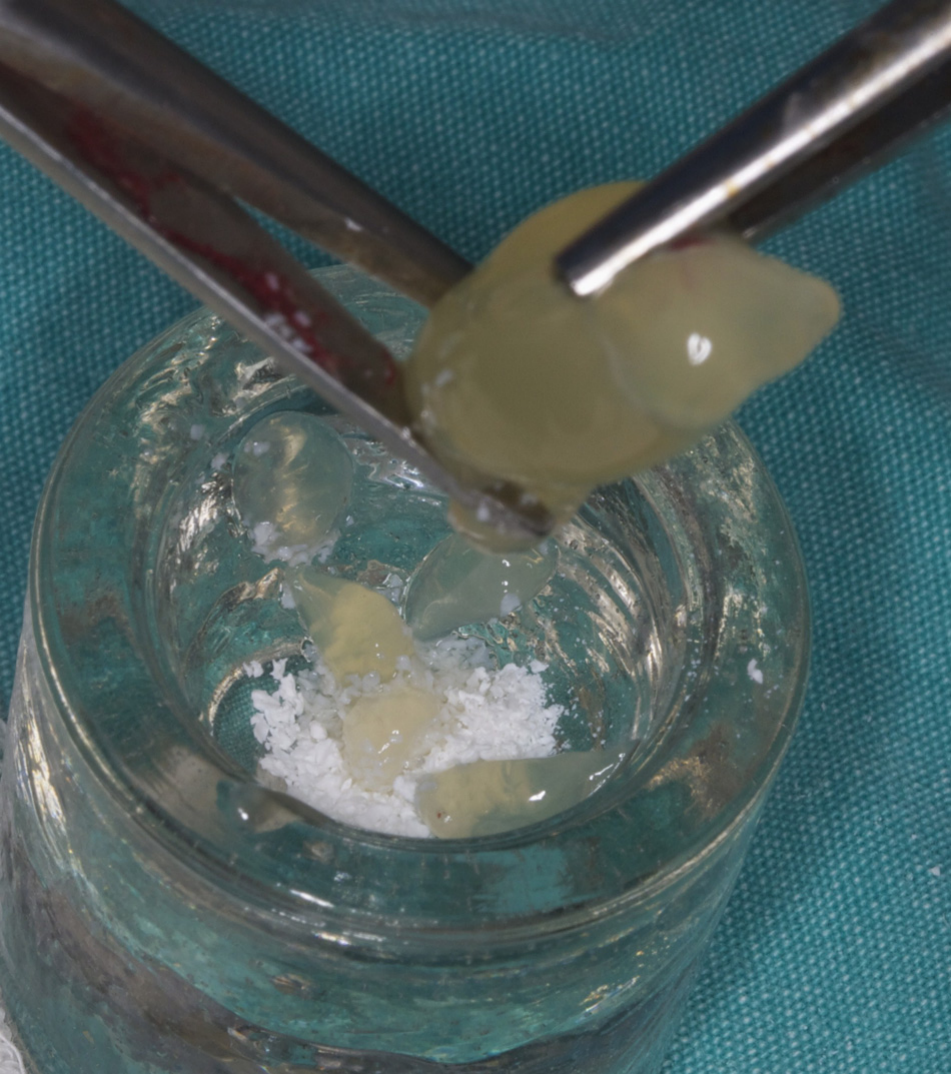
Fibrin clot (platelet-rich fibrin) was cut into small pieces and mixed with grafting material.

PRF was prepared as described by Choukroun et al.^1^ After administration of general anesthesia, venous blood was drawn from the jugular vein into 10-mL tubes (Vacuette, Grenier Bio-One, Kremsmünster, Austria) without anticoagulant. A total of 80 mL of blood was taken from each animal. The tubes were immediately centrifuged at 400*g* for 12 minutes (Process, Nice, France). After centrifugation, 3 layers were obtained: acellular plasma (platelet poor plasma) was concentrated at the top and was collected by syringe (Figure 3); fibrin clots and red corpuscles were removed from the tube with a scalpel (Figure 4); a PRF clot was immediately separated from red corpuscles by tweezers. This clot was either cut into small pieces and mixed with graft material or pressed between 2 sterile compresses to obtain a membrane (Figure 5).

### Histologic and histomorphometric examination

Histopathologic and histomorphometric examinations were performed by 1 blinded examiner (M.T.S.). All harvested specimens were fixed in 10% buffered formalin for 1 week. After fixation, all specimens were treated with 50% formic acid and 20% sodium citrate solution for decalcification. The decalcified specimens were embedded in paraffin and cut into 3-µm-thick sections on charged slides using a microtome (Leica Microsystemic RM 2125, Leica, Berlin, Germany), and routine hematoxylin and eosin staining was performed. The cutting line was started parallel to the transverse axis of tibias at ∼4 mm away from tibia heads, where the first defect preparation was made. The sections were examined with a light microscope (Olympus BX60, Tokyo, Japan). Histopathologic images were captured at 10× and 20× magnification using the microscope connected to a digital camera (Olympus E-330) connected to a computer. Fibrosis, inflammation, and necrosis were evaluated. The relative inflammatory intensity was scored as follows: 0 = no inflammation; 1 = minimal inflammation; 2 = moderate inflammation; 3 = severe inflammation. For histomorphometric examination, 4 fields were captured at 10× magnification from each histopathologic section. The pictures were transferred to the imaging program (Olympus Soft Imaging System Analysis). The proportions of the area occupied by newly formed bone (NB) and residual graft materials (RG) were measured. All measurements were confined to a total area of 0.38 mm^2^.

#### Statistical analysis

Statistical analyses were performed using SPSS for Windows (Version 10; SPSS Inc, Chicago, Illinois). Data are expressed as numbers or mean (SD). The Kruskal-Wallis test was used to compare the 4 groups at days 10, 20, and 40 in terms of inflammation, new bone formation, and residual bone graft. The Mann-Whitney *U* test was used for pairwise comparisons of groups that showed significant a difference from the Kruskal-Wallis test.

Throughout the study period, overall differences between the 4 groups were assessed with Pillai’s Trace test for repeated measures of the general linear model, and Bonferroni correction was used for pairwise comparison. Differences were considered significant at a *P* value of ≤0.05.

## Results

Healing was uneventful in all animals. No complications were observed. A total of 48 samples were evaluated.

**Fig. 6:**
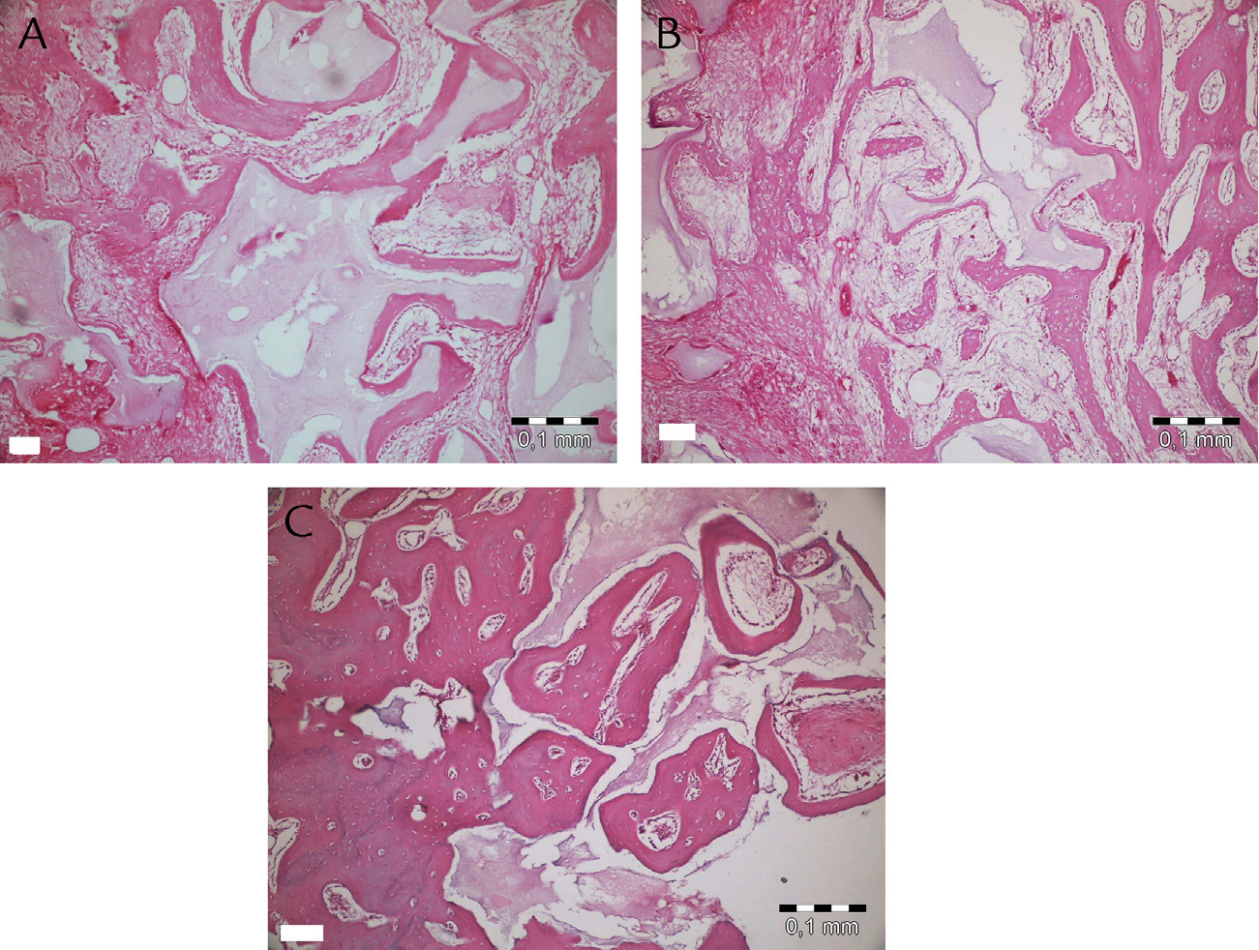
Histopathologic evaluation of platelet-rich fibrin + graft at 10 (A), 20 (B), and 40 (C) days.

Figures 6 through 9 show the histopathologic examination of the groups at 10, 20, and 40 days at 10× or 20× magnification.

None of the groups displayed any signs of necrosis. In the empty and PRF groups, fibrous tissue was observed distinctly at 10 days. The PRF+BCP and BCP groups showed fibrosis around the reduced graft material, surrounded by new bone formation. Active fibrosis tissue involving primary mesenchymal cells around new bone trabecula and graft material was observed in all animals at 20 and 40 days. Diagnosed fibrosis tissue gradually reduced by day 40 and was replaced by new bone tissue. New bone tissue was observed to be connected with graft material at 10 and 20 days, and apposition lines of material were observed to be different. At day 40, bone tissue was found to be mature, especially in surface areas of defects, and to have a cortical bone appearance. New bone formation in trabecular structure was observed around graft material present in the apical sides of the defect. Osteoclastic activity was observed both in new bone tissue and graft tissue.

Inflammation was observed in all groups at 10 days. Only the empty defects (all) and 2 specimens from the PRF+BCP group showed inflammatory cell infiltration at 20 days. At 40 days, inflammation was not observed in any group. Table I shows mean inflammation intensity scores. Kruskal-Wallis analysis showed a nonsignificant difference at day 10 (χ^2^ = 5.90, *P* = 0.12) and a significant difference at day 20 (χ^2^ = 11, *P* = 0.01). Mean (SD) values showed no difference between empty and PRF+graft groups (*Z* = −1.53, *P* = 0.13).

Kruskal-Wallis analysis showed that the new bone ratio at each time point was significantly different between groups. Table II shows NB for all groups and time intervals. At 10 days, the lowest amount of NB (3.4 [0.7]) was observed in empty defects, which was significantly less than in the PRF and PRF+BCP groups (*Z* = −2.18, *P* = 0.03 and *Z* = −2.30, *P* = 0.02, respectively) and nonsignificantly less than BCP (*Z* = −1.60, *P* = 0.11). The mean NB value was nearly the same in the PRF and BCP groups (7.4 [0.7%] and 7.2 [1.6%], respectively; Z = −0.44, *P* = 0.66). Statistical analysis showed that the defects filled with PRF+BCP mixture had statistically higher NB (11.4 [0.7%]) than PRF- and BCP-treated defects (*Z* = −2.31, *P* = 0.02; Z = −2.31, *P* = 0.02, respectively).

In the PRF+BCP group, NB occupied 42.2 (0.9)% of the defect volume at 20 days period (Table II). The other biomaterials and the empty defects showed similar patterns (empty defect, 24.9 [0.8]%; PRF, 29.5 [1.6]%; BCP group, 29.6 [1.7]%). The statistical analysis determined the significance between empty defects and PRF and between empty defects and BCP (*Z* = −2.32, *P* = 0.02 and *Z* = −2.07, *P* = 0.04, respectively). No difference was found between the defects treated with either PRF or BCP (*Z* = −0.29, *P* = 0.77).

At 40 days, the defects in the BCP and PRF+BCP groups were approximately half filled with new bone (49.1 [3.1]% and 54.9 [0.8]%, There was no significant difference between these 2 groups (*Z* = −1.60, *P* = 0.11). The defects treated with PRF+BCP showed more NB than the other 2 groups (*Z* = −2.32, *P* = 0.02 and *Z* = −2.19, *P* = 0.03 for empty and PRF groups, respectively). The empty defect group (39.7 [3.1]%) showed values close to those of the PRF (38.9 [4.9]%) and BCP (49.1 [3.1]%) groups, and there was no significant difference between these groups (*Z* = −0.15, *P* = 0.89 and *Z* = −1.73, *P* = 0.08, respectively). As the 2 other time intervals, the PRF and BCP groups did not show any difference (*P* = 0.11).

During the 40-day evaluation period, the PRF+BCP group showed the highest NB ratios, whereas the other 3 groups showed statistically similar results. Table III shows the results for the Pillai Trace test for repeated measures of the general linear model.

Table IV shows the mean and SD of RG materials in BCP and PRF+BCP groups. In both groups, the RG ratios were decreased at subsequent evaluation periods. The differences between 2 groups were not statistically significant at 10, 20, and 40 days (*P* = 0.25, *P* = 0.25, *P* = 0.66, respectively) and also during the 40-day follow-up (*P* = 0.26).

## Discussion

Various bone substitutes have been introduced for ridge preservation, bone augmentation, and filling peri-implant defects in oral implantology. Among the variety of grafting materials, PRF has become a focus of current studies due to its potential to accelerate and improve the healing process. In the present study, we evaluated PRF in combination with BCP in surgically created experimental bone defects. The results showed more new bone formation in defects filled with PRF+BCP than BCP alone, PRF alone, and empty defects. Also, no differences were observed in RG values between the PRF+BCP and BCP groups.

Each bone substitute has advantages and disadvantages. Autogenous bone grafts are still considered to be the gold standard because of their osteogenic, osteoconductive, and osteoinductive properties. Nevertheless, this type of augmentation has the disadvantages of requiring for a second operative donor site and having rapid resorption. Allogenic and xenografts grafts are produced from other humans or animals, making these materials unacceptable to some patients. These materials also involve the risk of carrying disease. Synthetic or alloplastic grafting products such as HA/TCP composite ceramics (BCP) therefore provide another option. The advantages of BCP compared with autogenous grafts are their synthetic origin, biocompatibility, osteoconductivity, unlimited quantity, and avoidance of a second surgical site. Therefore, clinically applied BCP was preferred in the study.

The literature includes few studies using only PRF or graft materials with different characteristics combined with PRF.

Tatullo et al^23^ conducted histological and clinical evaluations of 60 patients who underwent sinus lifting surgery before implant surgery. The experimental group received bovine bone graft material (Bio-Oss) combined with PRF, whereas the control group received only bovine bone graft material (Bio-Oss, Geistlich Pharma AG, Wolhusen, Switzerland). The study made histologic and histomorphometric evaluations on days 106, 120, and 150. The results revealed that the good osteoconductive capacity of PRF led to the production of new bone, even at 106 days. No implant loss was observed at 36 (10) months. Primary implant stability, assessed by means of resonance frequency analysis, did not show any statistical difference between test and control groups. Ozdemir et al^9^ assessed the effects of PRF on bone augmentation in an animal model. Surgically created defects were filled with PRF, BCP, or anorganic bovine bone (ABB) and were covered with titanium membranes. Control groups were left empty. Histomorphometric evaluation was carried out at 1 and 3 months. The control group showed the least new bone formation, and similar new bone areas were found among PRF, BCP, and ABB groups after 1 month. PRF and ABB showed a greater area of new bone formation than the other 2 groups at 3 months. Contrary to the results of these studies, we observed statistically higher NB in the group with defects filled with PRF+BCP than the other groups on days 10 and 20. Also, at 40 days, there was relatively similar NB in all 4 defects. These results prove that PRF is effective in the early stages of healing. Our results were confirmed by the studies of Zhang et al^32^ and Choukroun et al.^27^ Zhang et al^32^ found no difference in NB and residual bone substitute between a group receiving only bovine bone graft (Bio-Oss) and a group receiving PRF in combination with bovine bone graft 6 months after sinus-lifting surgery. Choukroun et al^27^ examined the efficacy of freeze-dried bone allograft compared with freeze-dried bone allograft combined with PRF. Histologic evaluation revealed that maturation in the PRF group at 4 months of healing was similar to that in the control group at 8 months.

Because of ethical reasons, relatively long-term studies have to be planned for clinical studies as the studies listed above. Although PRF has longer resorption and remodeling times than the other described platelet concentrations,^33^ it has features to similar to those of natural clotting. Therefore, experimental evaluations investigating the early stages of healing would be beneficial.

Kim et al^34^ used TCP, PRF+TCP, and recombinant human bone morphogenic protein 2 (rhBMP-2)–coated TCP in the augmentation of the maxillary sinus in rabbits. The animals were killed at 3 days and at 1, 2, 4, 6, and 8 weeks. The PRF+TCP group showed greater area of bone formation compared with the TCP and the rhBMP-2–coated TCP groups during the evaluation period. In contrast to this study, the present study used sheep, which have a blood structure similar to that in humans, which allowed creation of sufficient defect capacity that would be clinically encountered and a histological examination to be conducted, especially in the first phases of recovery. Therefore, this study used the same PRF preparation technique as that used in humans.

## Conclusions

Within the limitations of this experimental study, it can be concluded that PRF in addition to BCP may favor the formation of new bone. The effectiveness of PRF depends not only on its features but also the properties of coadministered grafting material. Although it is reported that PRF dissolves more slowly than other platelet concentrates, it does not exceed months when the clinical samples only can be collected; therefore, further experimental studies are necessary to investigate the effects of PRF and coadministered biomaterials on bone healing.

## Conflicts of Interest

The authors have indicated that they have no conflicts of interest regarding the content of this article.

## Figures and Tables

**Fig. 7:**
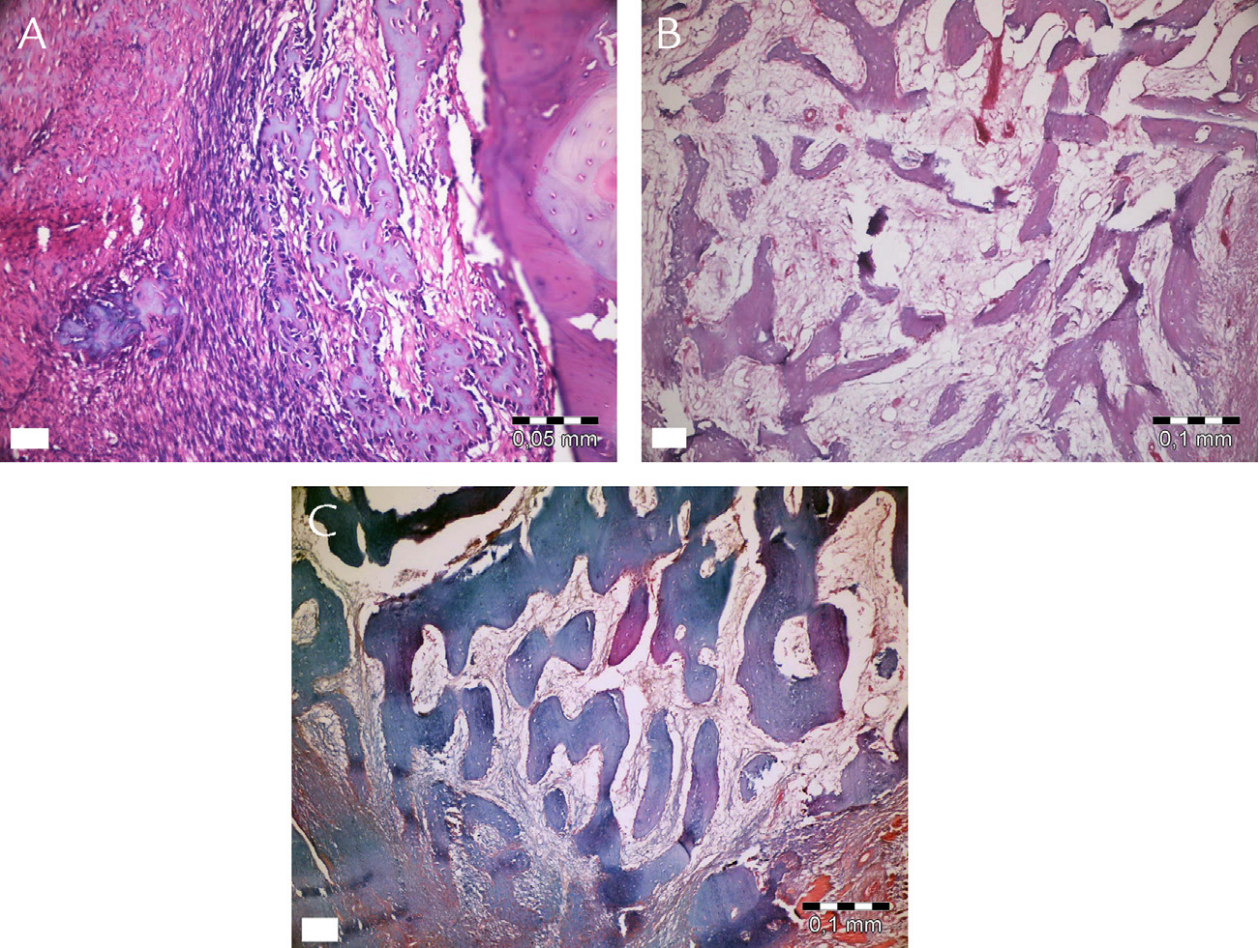
Histopathologic evaluation of platelet-rich fibrin at 10 (A), 20 (B), and 40 (C) days.

**Fig. 8:**
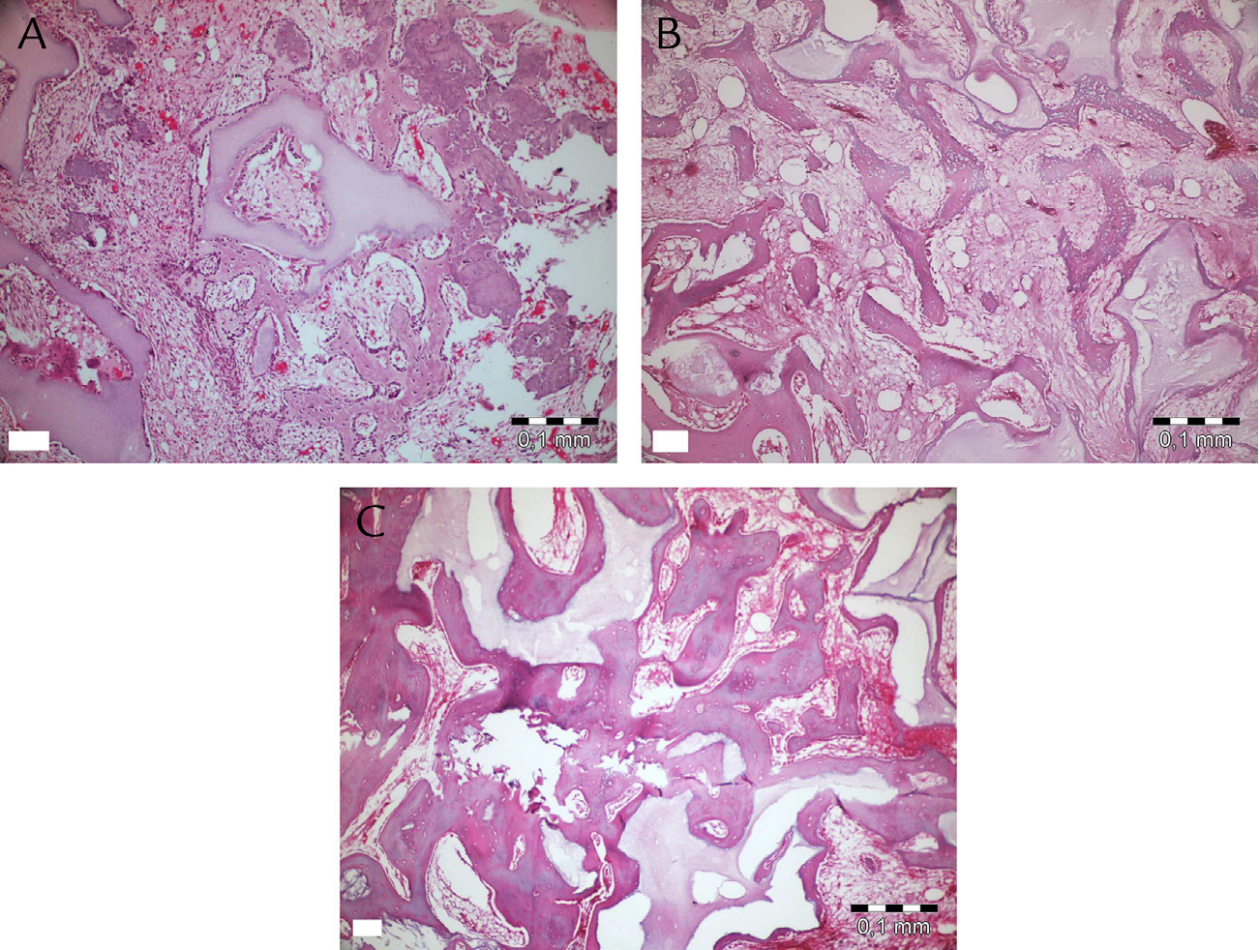
Histopathologic evaluation of biphasic calcium phosphate at 10 (A), 20 (B), and 40 (C) days.

**Fig. 9:**
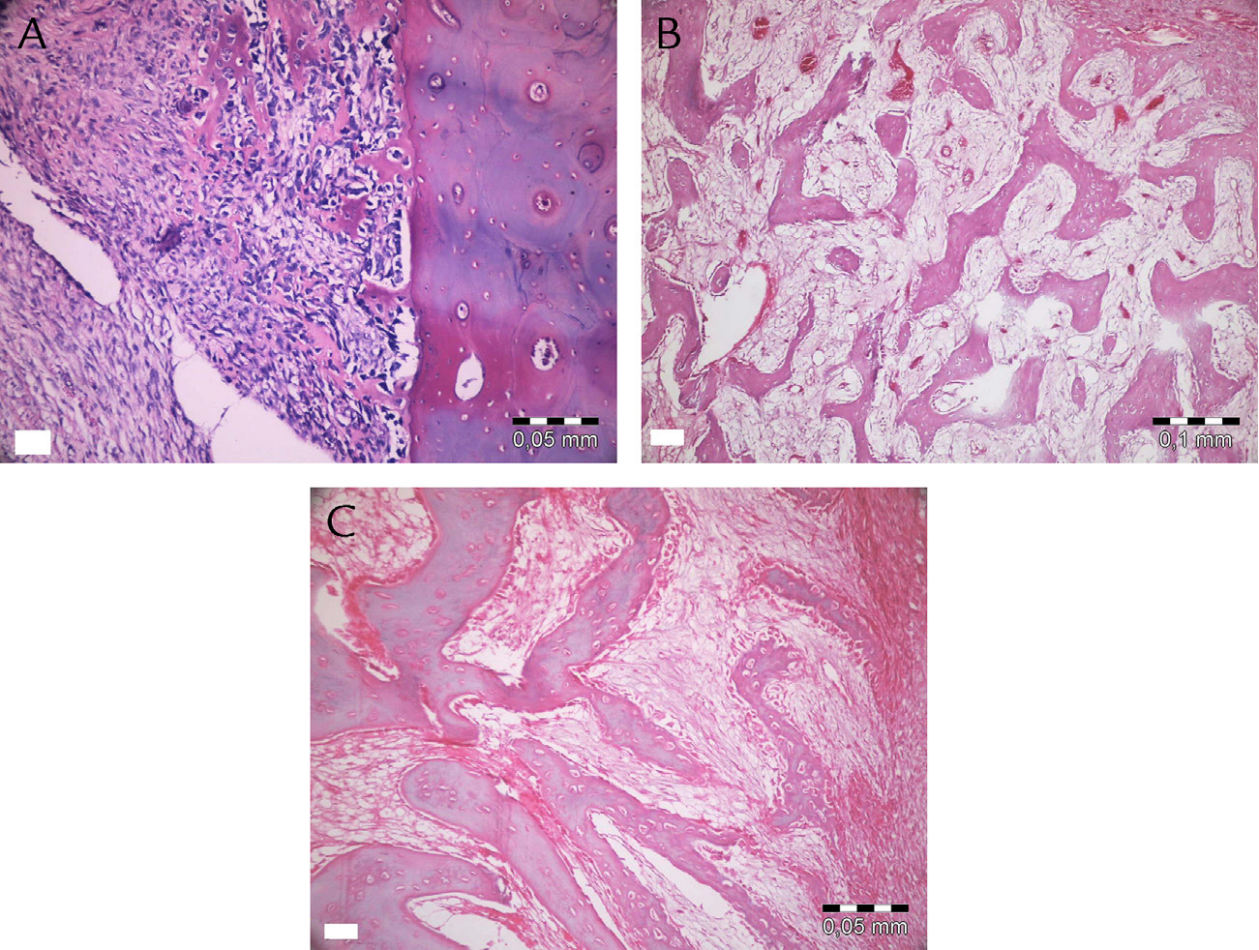
Histopathologic evaluation of empty defect at 10 (A), 20 (B), and 40 (C) days.

**Table I:** Inflammation intensity of 4 groups.

**Group**	**10 days**	**20 days**
Empty	1.5 (0.3)	1.0 (0.0)
PRF	1.0 (0.0)	0
Graft	1.0 (0.6)	0
PRF+graft	2.0 (0.0)	0.5 (0.3)

PRF, platelet-rich fibrin. The values are mean (SD). No inflammation was observed in any groups at 40 days.

**Table II:** New bone formation ratios in each group at 10, 20, and 40 days. Data are mean (SD).

Killing day	Empty defect	PRF	BCP	PRF+BCP
10 days	3.4 (0.7)	7.4 (0.7)	7.2 (1.6)	11.4 (0.7)
20 days	24.9 (0.8)	29.5 (1.6)	29.6 (1.7)	42.2 (0.9)
40 days	39.7 (3.1)	38.9 (4.9)	49.1 (3.1)	54.9 (0.8)

BCP, biphasic calcium phosphate; PRF, platelet-rich fibrin.

**Table III:** Pairwise comparison of groups for 40-day evaluation period.

Groups		*P*
Empty defect	PRF	1.0
	BCP	0.04
	PRF+BCP	0.001
PRF	Empty defect	1.0
	BCP	0.67
	PRF+BCP	0.001
BCP	Empty defect	0.04
	PRF	0.67
	PRF+BCP	0.012
PRF+BCP	Empty defect	0.001
	PRF	0.001
	BCP	0.012

BCP, biphasic calcium phosphate; PRF, platelet-rich fibrin.

**Table IV:** Residual bone substitute ratios in each group at 10, 20, and 40 days.

	**BCP**	**PRF+BCP**	***P***
10 days	30.8 (3.7)	22.1 (4.9)	0.248
20 days	17.8 (0.4)	19.5 (1.3)	0.245
40 days	11.6 (0.8)	10.9 (0.8)	0.661

BCP, biphasic calcium phosphate; PRF, platelet-rich fibrin.

## Footnotes

^⁎^ Trademark: Rompun^®^ (Bayer, Istanbul, Turkey).

^†^ Trademark: Ketalar^®^ (Eczacıbaşı, Istanbul, Turkey).
